# First trimester antenatal care contact in Africa: a systematic review and meta-analysis of prevalence and contributing factors

**DOI:** 10.1186/s12884-023-06034-1

**Published:** 2023-10-19

**Authors:** Ritbano Abdo, Minychil Demelash, Abdulrezak Mohammed Seid, Abdulhakim Mussema

**Affiliations:** 1 Department of Midwifery, College of Medicine and Health Sciences, Wachemo University, Hossana, Ethiopia; 2 Department of Medical Laboratory, College of Medicine and Health Sciences, Wachemo University, Hosanna, Ethiopia

**Keywords:** Antenatal care, Pregnant women, First-trimester antenatal care, Africa

## Abstract

**Background:**

Early detection, prevention, and management of diseases associated with pregnancy and pregnancy-related conditions depend on the beginning of antenatal care contact in the first trimester. Across Africa, regional and national differences are observed in the proportion of first-trimester ANC contact and the factors contributing to it. To create a suitable intervention plan, it is crucial to overcome these differences through single standard and uniform guidelines. This can be achieved through meta-analysis and systematic reviews. Therefore, this systematic review aimed to assess the pooled prevalence of first trimester ANC contact and the factors contributing to it in Africa.

**Methods:**

Observational studies conducted in Africa were retrieved from PubMed, Google Scholar, EMASE, CINHAL, Cochrane Library, Hinari databases and Mednar using combinations of search terms with Boolean operators. The JBI 2020 Critical Appraisal Checklist was used to assess the methodological quality of the studies. To assess publication bias, a funnel plot and Egger’s test were used to and I-squared was used to check the heterogeneity of the included studies. Data were extracted using Microsoft Excel and exported to Stata 16 software for analysis.

**Results:**

A total of 86 articles with 224,317 study participants from 19 African countries were included. The overall pooled prevalence of first-trimester ANC contact was 37.15% (95% CI: 33.3–41.0; I^2^ = 99.8%). The following factors were found to be significantly associated with first-trimester ANC contact: urban residence (OR = 2.2; 95% CI: 1.5–3.1; I^2^ = 98.5%); women under the age of 25 (OR = 1.5; 95% CI: 1.2–1.9; I^2^ = 94.1%);, educational status (OR = 1.8; 95% CI: 1.4–2.2; I^2^ = 96.1%), primiparity (OR: 1.7; 95% CI: 1.2–2.4: I^2^ = 97.4%), having planned pregnancies (OR: 2.1; 95% CI: 1.5–2.7; I^2^ = 95.5%) and employed women (OR = 1.7; 95% CI: 1.7–2.1; I^2^ = 94.4%).

**Conclusion:**

Because so few women in Africa initiate first-trimester ANC contact, it is clear that increasing maternal healthcare service uptake is still a challenge and will require significant effort to scale up the services. When working to improve maternal health in Africa, each nation's government and nongovernmental organizations should prioritize raising women's educational levels and providing pertinent information to rural women, focusing on reducing unintended pregnancies, women who live far from health facilities, women with low socioeconomic statuses, multiparous women and older women.

**Trial registration:**

PROSPERO International Prospective Register of Systematic reviews (ID: CRD42023401711).

**Supplementary Information:**

The online version contains supplementary material available at 10.1186/s12884-023-06034-1.

## Background

Despite considerable progress in maternal health over the last 20 years, there were still 287,000 maternal deaths worldwide in 2020. Over 99% of these deaths took place in low- and middle-income countries. Sub-Saharan Africa remains the region with the highest number of maternal deaths and accounts for approximately 70% of all maternal deaths reported globally in 2020 [1]. Western Africa reported the highest maternal deaths due to complications during and following pregnancy and childbirth, followed by Central and Eastern Africa. South Sudan, Chad and Nigeria recorded the highest maternal deaths during this period [2]. Most of these deaths were preventable with appropriate interventions during and following pregnancy and childbirth, but suitable solutions are not available, accessible or implemented in low-resource countries [1–3].

As evidence indicates, quality antenatal care (ANC) is a critical component of maternal healthcare services to reduce the unacceptable rate of maternal deaths [4, 5]. The World Health Organization(WHO) recommends at least eight contacts between a pregnant woman and a healthcare provider to enhance positive maternal outcomes and decrease perinatal mortality, with the first follow-up taking place within the first 12 weeks of gestation [5].

Early initiation of ANC contact in the first trimester can reduce maternal and neonatal mortality and improve maternal and newborn health [6]. First-trimester ANC contact is crucial for the early detection, prevention and treatment of pregnancy-related problems and concurrent diseases. It also establishes effective communication and provides timely information to pregnant women and their accompaniers, offering emotional and psychological support. Additionally, it allows for early reproductive health service integration [4, 7–9]. Furthermore, women can learn from healthcare practitioners about healthy practices throughout pregnancy, better understand warning signs during pregnancy and childbirth, and receive social, emotional, and psychological support at this vital time in their lives [4, 5, 7, 10, 11]. Moreover, it provides adequate time for suitable preparation for birth and emergencies and is an important ANC intervention that can prevent preventable maternal and perinatal mortality and morbidity.

Research has also shown that beginning ANC contact in the first trimester reduces perinatal care costs [12], as well as the high percentage of stillbirths [13], low birth weight and premature birth [14] that occur during the prenatal period. Moreover, first-trimester ANC contact enhances all-component care along the continuum of maternity, neonatal, child and reproductive healthcare services when completed in its entirety [9, 15–20]. It provides a crucial foundation for comprehending how maternal, infant and child health are interconnected [17, 18, 20].

Worldwide, the early ANC initiation rate is 58.6%, but it varies according to continent. In developing regions, the estimated rate of early antenatal care visits is 48.1%, compared to 84.8% in developed regions [21]. In Sub-Saharan Africa (SSA), a study conducted in 2022 found that the beginning of ANC visits during the first trimester is 38.0%. In Africa, first-trimester antenatal contact varies from country to country, ranging from 14.5% in Mozambique to 63.4% in Comoros [22]. Furthermore, previous studies have identified early ANC initiation as being associated with younger women [23], lower parity [24], higher socioeconomic status [25], secondary and high education [21, 25–28], planned pregnancy [10, 21–23, 26], knowledge about the correct time of ANC booking [10, 23, 25, 26, 29], distance from health facilities [23] and access to media [29].

As the evidence indicates, several primary studies were conducted in African countries to determine the prevalence of timely ANC initiation and its contributing factors [23, 25, 27, 28, 30–32]. Findings from these studies showed that prevalence and its contributing factors varied between and within countries. Although several researchers operationalized the initiation of early ANC before or at 16 weeks of gestation, this is contrary to the WHO's 2016 recommendation [6, 33–37]. Understanding both the contributing factors and prevalence of first-trimester ANC initiation, as recommended by the WHO, is essential for designing suitable maternal and perinatal guidance to improve maternal and unborn baby health during the perinatal period. Identifying the single figure and the common factors is critical for the development of effective initiatives that will improve maternal, newborn, and child healthcare service utilization in Africa. To address this issue, systematic and meta-analysis methods are appropriate. The purpose of this study was to assess the pooled prevalence of first-trimester ANC contact and its contributing factors in Africa.

## Methods

### Study protocol and reporting

The Preferred Reporting Items for Systematic Reviews and Meta-Analyses (PRISMA) guidelines were followed to conduct this systematic review and meta-analysis [38]. The eligibility criteria were adapted from the JBI 2017 review guidelines [39]. The review protocol was developed based on PRISMA 2020, submitted, and published in PROSPERO International's prospective register of systematic reviews (ID:CRD42023401711). We used Endnote (version X8) reference management software to download, organize, and review and Zoter to cite related articles.

## Inclusion criteria

### Study area: Africa

*Study participants*: Women aged 15 – 49 years (studies conducted on pregnant women or women who have delivered at least once in health facilities) were included. *Types of studies*: Observational Cross–sectional and case–control studies were considered. *Outcome of interests*: The primary studies reported the prevalence of first-trimester ANC contact and/or contributing factors of first-trimester initiation of ANC contact. *Publication condition*: Published and unpublished articles were included.

*Language*: English language. *Publication date*: Studies were conducted in Africa from January 2016, to March 2023 as recommended by the World Health Organization (WHO) in 2016, that every woman should initiate their first antenatal contact in the first trimester (within twelve of gestation).

## Exclusion criteria

Articles without an abstract and/or full text, duplicate studies, anonymous reports and qualitative studies were excluded from the analysis. In addition, studies that did not include outcomes in both the exposed and non-exposed groups were excluded after at least two email contacts with the primary author. These studies were excluded due to the inability to extract data from them in the absence of hard data. Furthermore, studies conducted in specific populations were excluded in order to make the studies included in the meta-analysis more similar with respect to all important variables. Moreover, depending on their quality score, one or more studies were eliminated since they used the same data source to prevent overlap.

## Variables and measures

*First-trimester ANC contact* is defined as the initiation of ANC contact in the first 12 weeks of gestation. *The place of residence* was classified as rural or urban *maternal age* was grouped into two categories: less than 25 years and 25 years or older and women's educational status was classified as secondary or above and below secondary. *Parity* was categorized as primiparous and multiparous. *Pregnancy status* is grouped into two categories: planned or unplanned (missed time or unwanted). *Wealth status* was classified into two categories: lowest (poor/poorest) and highest (rich/richest). *Occupational status* was categorized as working outside the home or working inside the home. The time *taken from home to health facilities* was categorized into two groups: less than an hour or more.

### Search strategy

We searched PubMed, Hinari, EMBASE, Cochrane, CINHAL, Google Scholar and Mednar databases to identify relevant studies. We also searched institutional university repositories for unpublished articles. Initially, we searched PubMed, Google and Google Scholar by article title to identify relevant key terms. Second, we identified similar idekeywords. Third, we searched the reference list of all identified reports and articles for additional studies and then searched the databases with these terms again. We used terms such as “Timely initiation of antenatal visit”, “first antenatal care visit”, “early antenatal care visit”, “early antenatal care initiation”, “first trimester antenatal care visit”, “associated factors”, “predictors”, “determinants”, “contributing factors”, “prevalence”, “magnitude”, “proportion”, “pregnant women” and “Africa”. We tested and refined with multiple test searches, andsimilar search terms were combined using Boolean operators such as OR, while different concepts were combined using Boolean operators such as AND (Additional file 1).

### Data extraction

MS Excel was used to extract the data. To gather the information required for analysis, two different data extraction formats were used. We included the last name of the author, the year of publication, the study country and region, the study design, sample size, the frequency of first trimester ANC contact, the prevalence with its confidence interval and the quality score of each study in the extraction form for prevalence. The data extraction format for contributing factors also contained the last name of the author, the publication year, the risk group (case and control), and the nonrisk group (case and control)**.** Two authors independently gathered all essential data, then cross-checked them and reached consensus on any differences.

### Quality assessment/critical appraisal

The searched article were exported manually into EndNote. Duplicates were removed, and the remaining articles were reviewed based on the inclusion and exclusion criteria. These criteria were tested on titles and abstracts to ensure their robustness in capturing articles related to first- trimester antenatal care contact in Africa. The Joanna Briggs Institute (JBI) quality appraisal checklist was used to evaluate the quality of individual studies [39]. Two reviewers independently assessed the quality of each primary study, and consensus was reached either to accept or reject each article based on the set criteria. When a disagreement occurred between the two reviewers, it was resolved by taking the mean score of the two reviewers. A quality was considered "low risk" if a study awarded > 50% of the quality assessment indicators. All identified cross-sectional studies were appraised using eight items: inclusion criteria, description of study subject and setting, valid and reliable measurement of exposure, objective and standard criteria used, identification of confounders, strategies to handle confounders, outcome measurement, and appropriate statistical analysis. All identified case–control studies were appraised using ten items: comparable groups, appropriateness of cases and controls, criteria to identify cases and controls, standard measurement of exposure, similarity in measurement of exposure for cases and controls, handling of confounder, strategies to handle confounder, standard assessment of outcome, appropriateness of duration for exposure, and appropriateness of statistical analysis. Finally, 88 cross-sectional studies and two case–control studies were evaluated, and 84 cross-sectional and two case–control studies received a quality score of 50% or above on the quality scale, indicating that they are low risk and were included in the analysis, while the remaining 4 studies received a high risk score and were excluded from the analysis (Additional file 2).

### Statistical analysis

Stata statistical software (version 16.0, StataCorp. LP, College Station, United States of America) was used to perform all analyses. A random effects meta-analysis model based on the DerSimonian and Laird approach was used to pool the prevalence and identify contributing factors of first trimester antenatal care contact in Eastern Africa. To declare statistical significance, a *p* value and 95% confidence interval were used. The random effect model was used for analyses with statistical heterogeneity, while the fixed effect model was used for analyses without heterogeneity. Statistical heterogeneity was checked using the I–squared (I^2^) statistic test [40]. The potential sources of heterogeneity were explored by meta-regression and subgroup analysis. Publication bias was viewed graphically by funnel plot asymmetry and tested through Egger’s [41]. The *p*-value was $<$ 0.05; there was statistical evidence for the presence of publication bias using the Egger’s test. Moreover, a counter-enhanced funnel plot was used to distinguish asymmetry due to publication bias or asymmetry from that due to other factors. Sensitivity analysis was performed to identify the effect of a single study on the overall estimate.

## Results

**Fig. 1:**
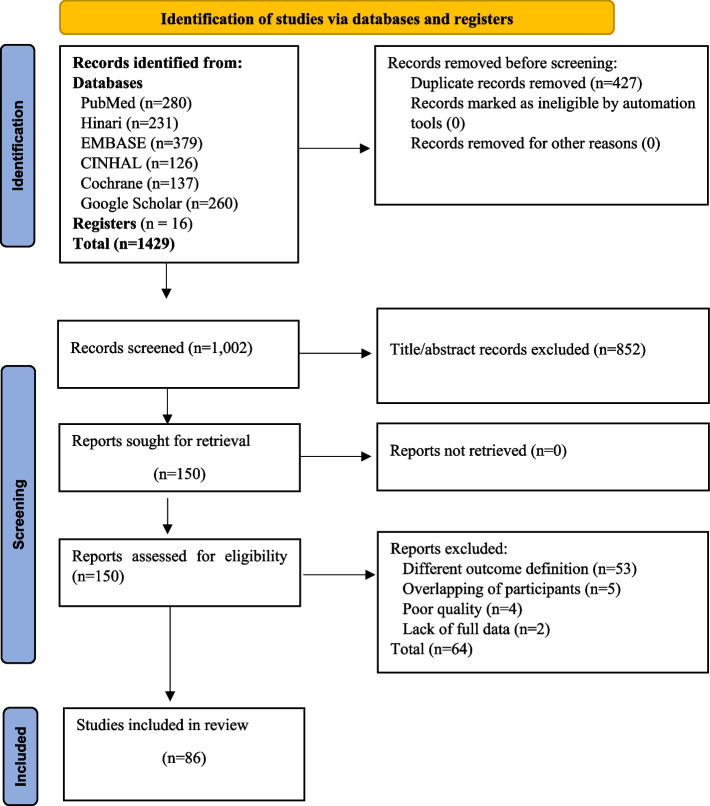
PRISMA flowchart diagram of the study selection process

A total of 1,429 studies (PubMed = 280, Hinari = 231, Cochrane Review = 137, CINHAL = 126, EMBASE = 379, Google Scholar = 260, other sources = 16), both published and unpublished were identified. Of all the articles, 427 were removed due to duplicates. Based on the inclusion and exclusion criteria of title and abstract selection, the eligibility of 1,002 abstracts was evaluated. The articles that did not fulfil the criteria were removed (*n* = 852), leaving a total of 150 articles for full text screening. A total of 150 articles were screened according to the eligibility criteria for full text selection. Furthermore, 53 articles were excluded due to researchers reporting different outcomes of interest, five due to overlapping study participants, four due to poor methodological quality and two due to a lack of full data. Finally, 86 studies were included in the meta-analysis. The same data source was used to prevent participant overlap, and one or more publications were eliminated based on the studies’ overall quality scores, with the highest-scoring research being included. Figure 1 shows the PRISMA flowchart summarizing the study selection process.

### Study characteristics

The final 88 studies were included from the 19 African countries included in our analysis. They included a study population of 224,317 women, of whom 76,255 initiated antenatal care in the first trimester of pregnancy. The majority of the studies were conducted in Eastern Africa (*n* = 56), followed by Western Africa (*n* = 19), Central Africa (*n* = 4), Northern Africa (*n* = 3) and Southern Africa (*n* = 2). Of the included articles, 86 were cross-sectional study designs, and two were case–control study designs. The sample sizes across the studies ranged from 140 [42] to 28,951 [43]. These studies represented the following countries: Ethiopia, Tanzania, Kenya, Uganda, Malawi, Rwanda, Zambia, Somalia, Egypt, Nigeria, Liberia, Ghana, South Africa, Guinea, Congo, Morocco, Cameroon, Sudan, Burkina Faso and Sierra Leone. We used 84 articles to compute the pooled prevalence of first-trimester ANC contact in Africa and two additional articles to identify the contributing factors involved (Table 1).

**Table 1:** Description of included studies in the meta-analysis

No	**Author name**	**Year**	**Country**	**Study sdesign**	**Region**	**Sample**	Frequency*	ES**[95%C]	**Quality**
1	Ekholuenetale et al. [36]	2020	Benin	cross-sectional	Western Africa	960	518	54.0[50.8, 57.0]	8
2	Some et al. [44]	2020	Burkina Faso	Cross-sectional	Western Africa	704	261	37.1[33.6, 40.7]	8
3	Samadoulougou et al. [45]	2018	Burkina Faso	Cross-sectional	Western Africa	9788	4144	42.3[41.4,43.3]	8
4	Appiah et al. [28]	2022	Cameroon	Cross-sectional	Central Africa	4183	1931	46.2[44.7, 47.7]	7
5	Njim [46]	2016	Cameroon	Cross-sectional	Central Africa	192	39	20.3[15.2, 26.6]	8
6	Tolefac et al. [47]	2022	Cameroon	Cross-sectional	Central Africa	293	164	56.0[ 50.3,61.5]	8
7	Myop et al. [48]	2022	Congo	Cross-sectional	Central Africa	1318	737	55.9[53.2, 58.6]	8
8	Halima et al. [49]	2021	Egypt	Cross-sectional	Northern Africa	160	96	60.0[52.3, 67.3]	7
9	Girma et al. [50]	2023	Ethiopia	cross-sectional	Eastern Africa	401	163	40.6[36.0, 45.5]	8
10	Wolde et al. [51]	2019	Ethiopia	cross-sectional	Eastern Africa	364	173	47.5[42.4, 52.7]	8
11	Jemberu et al. [52]	2020	Ethiopia	cross-sectional	Eastern Africa	421	107	25.4[21.5, 29.8]	8
12	Adere et al. [53]	2020	Ethiopia	Cross-sectional	Eastern Africa	390	158	40.5[35.8, 45.5]	8
13	Azeze et al. [54]	2020	Ethiopia	Cross-sectional	Eastern Africa	277	105	37.9[32.4, 43.7]	7
14	Tesfu et al. [23]	2021	Ethiopia	Cross-sectional	Eastern Africa	804	355	44.2[40.8, 47.6]	8
15	Debelo et al. [55]	2022	Ethiopia	Cross-sectional	Eastern Africa	330	137	41.5[36.3, 46.9]	7
16	Alene et al. [56]	2021	Ethiopia	cross-sectional	Eastern Africa	820	258	31.5[28.4, 34.7]	7
17	Shiferaw et al. [57]	2021	Ethiopia	Cross-sectional	Eastern Africa	1855	503	27.1[25.1, 29.2]	7
18	Yezengaw [58]	2022	Ethiopia	cross-sectional	Eastern Africa	207	136	65.7[59.0, 71.8]	7
19	Kolola et al. [59]	2020	Ethiopia	Cross-sectional	Eastern Africa	384	153	40.6[35.8, 45.6]	7
20	Ejeta et al. [60]	2017	Ethiopia	Cross-sectional	Eastern Africa	421	78	18.5[15.1, 22.5]	8
21	Wolde et al. [61]	2018	Ethiopia	Cross-sectional	Eastern Africa	416	169	40.6[36.0, 45.4]	8
22	Gidey et al. [62]	2017	Ethiopia	Cross-sectional	Eastern Africa	228	95	41.7[35.5, 48.2]	7
23	Tufa G et al. [63]	2020	Ethiopia	cross-sectional	Eastern Africa	377	218	57.8[52.8, 62.7]	7
24	Abebe et al. [64]	2023	Ethiopia	Cross-sectional	Eastern Africa	2935	1098	37.4[35.7, 39.2]	8
25	Tola et al. [65]	2021	Ethiopia	Cross-sectional	Eastern Africa	389	117	30.1[25.7, 34.8]	8
26	Adulo et al. [66]	2022	Ethiopia	Cross-sectional	Eastern Africa	2796	872	31.2[29.5, 32.9]	7
27	Kondale et al. [67]	2016	Ethiopia	Cross-sectional	Eastern Africa	225	61	27.1[21.7, 33.3]	6
28	Geta et al. [68]	2017	Ethiopia	Cross-sectional	Eastern Africa	608	132	21.7[18.6, 25.2]	8
29	Yaya et al. [69]	2017	Ethiopia	Cross-sectional	Eastern Africa	10,896	3672	33.7[32.8, 34.6]	8
30	Redi et al. [31]	2023	Ethiopia	Cross-sectional	Eastern Africa	375	157	41.9[37.0, 46.9]	8
31	Grum et al. [70]	2018	Ethiopia	Cross-sectional	Eastern Africa	614	88	14.3[11.8, 17.3]	8
32	Teshale et al. [71]	2020	Ethiopia	Cross-sectional	Eastern Africa	4741	1550	32.7[31.4, 34.0]	8
33	Tessema et al. [72]	2023	Ethiopia	Cross-sectional	Eastern Africa	344	118	34.3[29.5, 39.5]	8
34	Dibabu et al. [73]	2021	Ethiopia	Cross-sectional	Eastern Africa	454	184	40.5[36.1, 45.1]	8
35	Abera et al. [74]	2023	Ethiopia	Cross-sectional	Eastern Africa	392	142	36.2[31.6, 41.1]	8
36	Alemu et al. [25]	2018	Ethiopia	Cross-sectional	Eastern Africa	400	187	46.8[41.9, 51.6]	8
37	Drammeh et al. [75]	2018	Gambia	Cross-sectional	Western Africa	238	30	12.6[9.0,17.4]	7
38	Kotoh et al. [27]	2019	Ghana	Cross-sectional	Western Africa	431	38	8.8[6.5, 11.9]	8
39	Manyeh et al. [76]	2020	Ghana	Cross-sectional	Western Africa	1076	617	57.3[54.4, 60.3]	7
40	Ziblim et al. [77]	2022	Ghana	Cross-sectional	Western Africa	222	62	27.9[ 22.4, 34.2]	6
41	Anaba et al. [78]	2022	Ghana	Cross-sectional	Western Africa	2163	1472	19.8[17.8, 21.9]	8
42	Amoako et al. [79]	2021	Ghana	Cross-sectional	Western Africa	212	91	42.9[36.5, 49.7]	6
43	Peprah et al. [80]	2022	Ghana	Cross-sectional	Western Africa	400	232	58.0[53.1, 62.7]	8
44	Seidu et al. [81]	2021	Guinea	Cross-sectional	Western Africa	4274	968	22.7[21.4, 23.93]	6
45	Muthoni et al. [30]	2022	Kenya	cross-sectional	Eastern Africa	198	137	69.2[62.4, 75.2]	6.5
46	Wekesa et al. [82]	2017	Kenya	Cross-sectional	Eastern Africa	279	55	19.7[15.5, 24.8]	6
47	Ochako et al. [83]	2018	Kenya	Cross-sectional	Eastern Africa	3569	558	15.6([4.5, 16.9]	6
48	Musaa et al. [84]	2022	Kenya	Cross-sectional	Eastern Africa	6948	3645	52.5[51.3, 53.6]	7
49	Blackstone et al. [85]	2019	Liberia	Cross-sectional	Western Africa	5348	3556	66.5[ 65.2, 67.7]	8
50	Ekholuenetale et al. [86]	2022	Liberia	Cross-sectional	Western Africa	4095	2955	72.2[70.8, 73.5]	8
51	Machika et al. [87]	2017	Malawi	Cross-sectional	Eastern Africa	386	111	28.8[24.5, 33.5]	6
52	Kuuire et al. [43]	2017	Malawi	Cross-sectional	Eastern Africa	28,951	2885	10.0(9.6, 10.3]	8
53	Nkoka et al. [88]	2018	Malawi	Cross-sectional	Eastern Africa	6413	1503	23.4(22.4, 24.5]	5.5
54	Housni et al. [89]	2017	Morocco	Cross-sectional	Northern Africa	283	223	78.8[73.7, 83.2]	5
55	Okunowo et al. [90]	2019	Nigeria	cross-sectional	Western Africa	380	151	39.7[34.9,44.7]	8
56	Aliyu et al. [91]	2017	Nigeria	Cross-sectional	Western Africa	20,467	5485	26.8[26.2, 27.4]	8
57	Utuk et al. [92]	2017	Nigeria	Cross-sectional	Western Africa	370	102	24.0[23.4, 24.7]	6
58	Fagbamigbe et al. [93]	2021	Nigeria	Cross-sectional	Western Africa	16,448	3948	27.6[23.3, 32.3]	7
59	Anyaka et al. [94]	2020	Nigeria	Cross-sectional	Western Africa	787	420	53.4[ 49.9, 56.8]	7
60	Harindimana et al. [95]	2019	Rwanda	Cross-sectional	Eastern Africa	8853	3207	36.2[35.2, 37.2]	8
61	Uwimana et al. [96]	2023	Rwanda	Cross-sectional	Eastern Africa	18,034	9364	51.9[51.2, 52.7]	7
62	Bagambe et al. [97]	2021	Rwanda	Cross-sectional	Eastern Africa	5944	3339	56.2]54.9, 57.4]	6
63	Sserwanja et al. [98]	2022	Rwanda	Cross-sectional	Eastern Africa	6302	3696	58.6[57.4, 59.9]	8
64	Innocent et al. [42]	2021	Rwanda	Cross-sectional	Eastern Africa	140	60	42.9[35.0, 51.1)	8
65	Sserwanja et al. [99]	2022	Sierra Leone	Cross-sectional	Western Africa	5432	2399	44.2[42.9, 45.5]	8
66	Hassen et al. [100]	2019	Somalia	Cross-sectional	Eastern Africa	247	149	60.3[54.1, 66.2]	8
67	Smith [98]	2019	South Africa	cross-sectional	Southern Africa	213	48	22.5[17.4, 28.60]	7
68	Haffejee et al. [101]	2018	South Africa	Cross-sectional	Southern Africa	329	35	10.6[7.8, 14.4]	6
69	Rahatamalleh et al. [102]	2017	Sudan	cross-sectional	Northern Africa	320	199	62.2[56.8, 67.3]	6
70	Moshi et al. [103]	2021	Tanzania	Cross-sectional	Eastern Africa	6924	1586	22.9[21.9, 23.9]	8
71	Njiku et al. [104]	2017	Tanzania	Cross-sectional	Eastern Africa	240	71	29.6[24.2, 35.6]	7
72	Katembo l [105]	2017	Tanzania	Cross-sectional	Eastern Africa	352	106	30.1[25.6, 35.1]	7
73	Kisaka et al. [106]	2020	Tanzania	Cross-sectional	Eastern Africa	311	91	29.3[24.5, 34.5]	7
74	Tungaraza et al. [107]	2022	Tanzania	Cross-sectional	Eastern Africa	384	88	22.9[19.0, 27.4]	6
75	Okello et al. [108]	2018	Uganda	Cross-sectional	Eastern Africa	390	227	58.2[53.3, 63.0]	8
76	Benah et al. [109]	2017	Uganda	Cross-sectional	Eastern Africa	156	56	35.9[28.8, 43.7]	5
77	Mazera et al. [110]	2019	Uganda	Cross-sectional	Eastern Africa	305	47	15.4 [11.8, 19.9]	7
78	Komuhangi et al. [111]	2020	Uganda	Cross-sectional	Eastern Africa	283	31	11.0[7.8, 15.1]	8
79	Towongo et al. [112]	2022	Uganda	Cross-sectional	Eastern Africa	9433	2802	29.7[28.8, 30.6]	7
80	Betty et al. [113]	2018	Uganda	Cross-sectional	Eastern Africa	216	14	6.5[3.9, 10.6]	6
81	Chewe et al. [114]	2016	Zambia	cross-sectional	Eastern Africa	305	41	13.4[10.1, 17.7]	6
82	Mwanamwambwa (2021) [115]	2021	Zambia	Cross-sectional	Eastern Africa	200	123	61.5[54.6, 68.0]	7
83	Mbaala et al. [116]	2019	Zambia	Cross-sectional	Eastern Africa	454	157	34.6[30.4, 39.1]	7
84	Sinyange et al. [117]	2016	Zambia	Cross-sectional	Eastern Africa	3979	756	19.0[17.8, 20.2]	5
**Over all pooled prevalence from random effect model**								**37.15[33.3,41.0]**	
**Additional two studies included in factor analysis**									
85	Wolderufael et al. [118]	2020	Ethiopia	Case–control	Eastern Africa	459	153	––	9.5
86	Gebrekidan et al. [119]	2017	Ethiopia	Case–control	Eastern Africa	402	268	––-	9.8

### Pooled prevalence of timely initiation of antenatal care in Africa

**Fig. 2:**
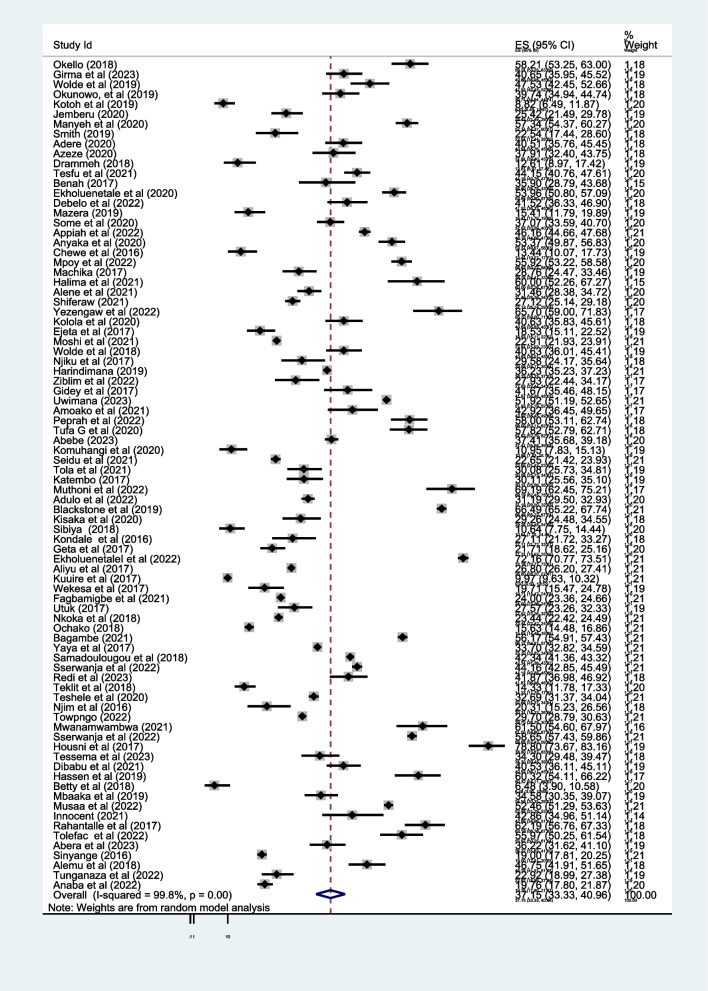
Overall pooled prevalence of first trimester ANC contact in Africa, 2023

The overall pooled prevalence of first-trimester ANC contact in Africa using the fixed effect model was 29.0% [95% CI: 28.8–29.1]. When using the fixed effect model, the pooled effect size of first-trimester ANC contact showed significant heterogeneity among the included studies *(*I^2^) of 99.8% (*P* = 0.00). As a result, we determined the final prevalence with a random-effect model to control the observed unevenness. The final pooled prevalence of first-trimester ANC contact was 37.15% [95% CI: 33.3, 41.0] (Fig. 2).

### Subgroup analysis by country and region

We performed subgroup analysis by country and region to handle heterogeneity. In addition, to identify possible sources of heterogeneity, regression with sample size and year of publication was performed. The analysis noted differences in prevalence between countries and regions. The subgroup analysis indicated that the prevalence of first-trimester ANC contact ranged from 20.6% in Malawi to 62.2% in Sudan. This study also showed that the prevalence of first-trimester ANC contact was 35.2%, 34.8%, 44.8%, 67.2% and 13.7% in studies in Eastern, Western, Central, Northern and Southern Africa, respectively (Table 2). Meta-regression analysis indicated that heterogeneity was explained by publication year (*P* = 0.001) (Table 3). It is well known that meta-analyses of prevalence frequently show high variation. As a result, true heterogeneity is expected in prevalence estimates due to differences in the time and place in which the included studies were conducted, which may not be discriminative and should be interpreted with caution in this case [120]. Due to a lack of power and precision, I^2^ estimates can be unreliable at times. High heterogeneity may be the result of the presence of time-dependent bias or sample size dependence [120, 121].

**Table 2:** Subgroup analysis of pooled prevalence of first trimester antenatal care contact in Africa, 2023

**Variable**	**Characteristics**	**NS**	**Pooled prevalence 95%CI**	I^2^	***P* value**
**Country**	Uganda	6	26.0[13.8, 38.2]	98.9	0.00
	Ethiopia	28	36.5[33.6, 39.3]	96.3	0.00
	Zambia	4	31.8[17.6, 46.0]	98.5	0.00
	Malawi	5	20.6[9.5, 31.7]	–	–
	Tanzania	5	27.5[22.8, 32.3]	77.8	0.00
	Rwanda	5	49.4[40.7, 58.0]	99.6	0.00
	Kenya	4	39.2[14.3, 64.1]	99.8	0.00
	Somalia	1	60.3[54.1, 66.2]	–	–
	Nigeria	5	33.9[29.0, 38.8]	98.8	
	Ghana	6	35.8[18.0, 253.4]	99.4	
	Cameroon	3	40.9[24.6, 57.2]	–	
	South Africa	2	13.7[10.9, 16.6]	–	
	Burkina Faso	2	42.0([41.0, 42.9]	–	
	Congo	1	55.9[ 53.2, 58.6]	–	
	Egypt	1	60.0[52.3, 67.3]	–	
	Guinea	1	22.7[21.4, 23.9]	–	
	Liberia	2	69.1[68.2, 70.0]	–	
	Sierra Leone	1	44.2[42.9, 45.5]	–	
	Sudan	1	62.2[56.8, 67.3]	–	
Region	Eastern Africa	56	35.6[31.8, 39.4]	99.5%	
	Western Africa	21	40.5[32.5, 48.5]	99.8%	
	Central Africa	4	44.8[34.1, 55.5]	87.8%	
	Northern Africa	3	67.2[54.8, 79.6]	–-	
	Southern Africa	2	13.7[10.9, 16.6]	–-

**Table 3:** Meta-regression analysis of factors affecting between-study heterogeneity

Source of heterogeneity	Coef	Std. Err	Z	*P* >|z|	[95% Conf. Interval]
Year of publication	2.868348	.8107	3.54	0.000	1.279406 4.457291
Sample size	-.0003462	.0003429	-0.01	0.313	-.0010184 .0003259

### Publication bias

**Fig. 3:**
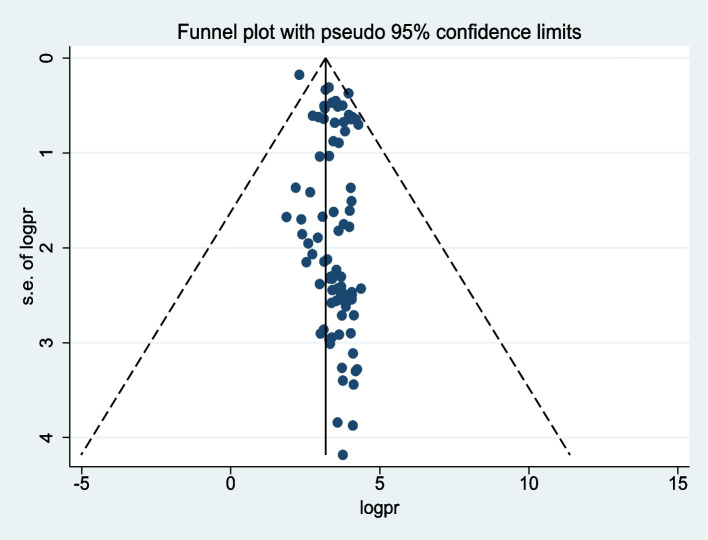
Funnel plot for publication bias for prevalence of first-trimester ANC contact in Africa

**Fig. 4:**
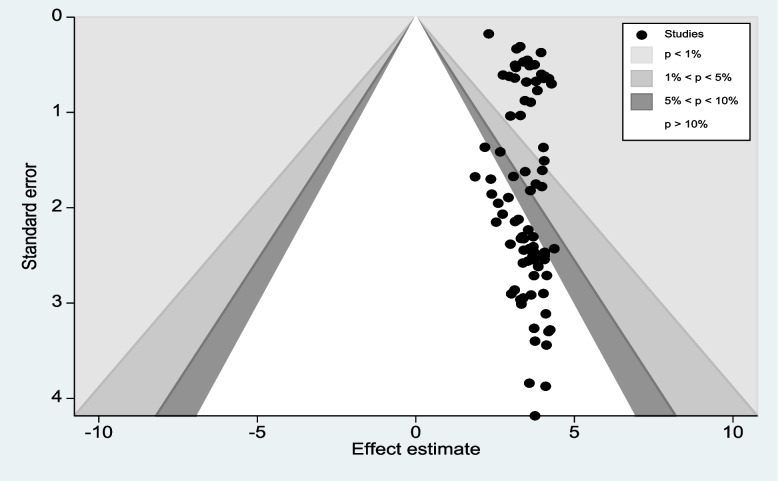
Counter enhanced funnel plots for publication bias for the prevalence of first-trimester ANC contact in Africa

**Fig. 5:**
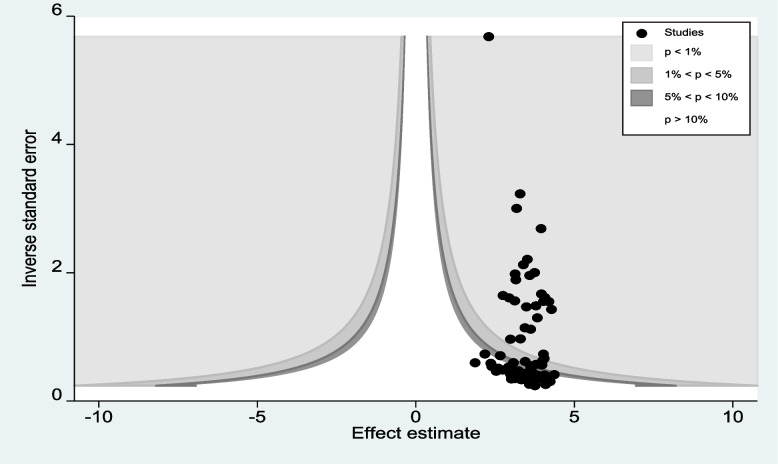
Meric inverse counter-enhanced funnel plots of publication bias for the prevalence of first-trimester ANC contact in Africa

A funnel plot and Egger’s regression test were used to assess publication bias. The funnel plot showed evidence of asymmetry (Fig. 3). Egger’s regression test was also statistically significant, with a *P* value of 0.001. However the funnel plot and Egger’s test only assess the risk of small study bias, and smaller studies tend to have greater variance. Sure, an asymmetrical plot might be due to publication bias, but there could be other reasons that explain funnel plot asymmetry. Therefore, we performed a counter-enhanced funnel plot to distinguish asymmetry due to publication bias or from that due to other factors. The contour-enhanced funnel plot indicates that the “missing” studies are expected to lie in areas of high statistical significance (the shaded area), whereas the majority of available studies are nonsignificant, indicating that the observed asymmetry may not be due to publication bias based on statistical significance. Thus, the asymmetry may have been caused by a number of other factors, such as study size, study effect, and study design (Fig. 4). Similar findings also occurred when we performed a metric (inverse) counter-enhanced funnel plot (Fig. 5).

### Sensitivity analysis

We performed a sensitivity analysis to investigate the impact of each individual study on the overall meta-analysis summary estimate. No studies were found to be outside the confidence bounds in the sensitivity analysis, implying that all studies had a nearly equal influence on the pooled prevalence (Table 4).

**Table 4:** Sensitivity analysis for first trimester antenatal care contact in Africa

Study omitted	Estimate	[95% Conf. Interval]	
Okello et al. (2021) [108]	37.0	33.3	40.8
Girma et al. (2023) [50]	37.2	33.5	41.0
Wolde et al. (2019) [51]	37.2	33.4	40.9
Wolde et al. (2018) [61]	37.2	33.4	40.9
Okunowo, et al. (2019) [90]	37.3	33.5	41.0
Kotoh et al. (2019) [27]	37.6	33.9	41.4
Jemberu (2020) [52]	37.4	33.7	41.2
Manyeh et al. (2020) [76]	37.0	33.3	40.8
Smith (2019) [98]	37.5	33.7	41.2
Adere (2020) [53]	37.2	33.5	41.0
Azeze (2020) [54]	37.3	33.5	41.0
Drammeh (2018) [75]	37.6	33.8	41.3
Tesfu et al. (2022) [23]	37.2	33.5	40.9
Benah (2027) [109]	37.3	33.6	41.0
Ekholuenetale et al. (2020) [36]	37.1	33.3	40.8
Debelo et al. (2022) [55]	37.2	33.5	41.0
Mazera (2016) [110]	37.5	33.8	41.3
Some et al. (2020) [44]	37.3	33.5	41.0
Appiah et al. (2022) [28]	37.2	33.4	40.9
Anyaka et al. (2020) [94]	37.1	33.4	40.8
Chewe et al. (2016) [114]	37.6	33.8	41.3
Mpoy et al. (2022) [48]	37.1	33.3	40.8
Machika (2017) [87]	37.4	33.6	41.1
Halima et al. (2021) [49]	37.0	33.3	40.8
Alene et al. (2021) [56]	37.4	33.6	41.1
Shiferaw (2021) [57]	37.4	33.6	41.2
Yezengaw et al. (2022) [58]	37.0	33.2	40.7
Kolola et al. (2020) [59]	37.2	33.5	41.0
Ejeta et al. (2017) [60]	37.5	33.8	41.2
Moshi et al. (2021) [103]	37.5	33.6	41.3
Wolde et al. (2018) [61]	37.2	33.5	41.0
Njiku et al. (2017) [104]	37.4	33.6	41.1
Harindimana (2019) [95]	37.3	33.5	41.1
Ziblim et al. (2022) [77]	37.4	33.6	41.1
Gidey et al. (2017) [62]	37.2	33.5	41.0
Uwimana (2023) [96]	37.1	33.5	40.8
Amoako et al. (2021) [79]	37.2	33. 5	41.0
Peprah et al. (2022) [80]	37.0	33.3	40.8
Tufa G et al. (2020) [63]	37.0	33.3	40.8
Abebe (2023) [64]	37.3	33.5	41.0
Komuhangi et al. (2020) [111]	37.6	33.8	41.3
Seidu et al. (2021) [81]	37.5	33.7	41.2
Tola et al. (2021) [65]	37.4	33.6	41.1
Katembo (2017) [105]	37.4	33.6	41.1
Muthoni et al. (2022) [30]	36.9	33.2	40.7
Adulo et al. (2022) [66]	37.4	33.6	41.1
Blackstone et al. (2019)| [85]	36.9	33.3	40.5
Kisaka et al. (2020)| [106]	37.4	33.6	41.1
Hoffejee (2018) [101]	37.6	33.8	41.3
Kondale et al. (2016) [67]	37.4	33.7	41.1
Geta et al. (2017) [68]	37.5	33.7	41.2
Ekholuenetale et al. (2022) [86]	36.9	33.3	40.4
Aliyu et al. (2017) [91]	37.4	33.5	41.3
Kuuire et al. (2017) [43]	37.6	34.4	40.8
Wekesa et al. (2018) [82]	37.5	33.7	41.2
Fagbamigbe et al. (2021) [93]	37.4	33.6	41.3
Utuk (2017) [92]	37.4	33.6	41.1
Nkoka et al. (2018) [88]	37.4	33.6	41.2
Ochako (2016) [83]	37.5	33.8	41.3
Bagambe (2021) [97]	37.1	33.4	40.8
Yaya et al. (2017) [69]	37.3	33.5	41.2
Samadoulougou et al. (2018) [45]	37.2	33.4	41.0
Sserwanja et al. (2022) [15]	37.2	33.4	41.0
Redi et al. (2022) [31]	37.2	33.5	41.0
Grum et al. (2018) [70]	37.6	33.8	41.3
Teshale et al. (2020) [71]	37.3	33.6	41.1
Njim et al. (2016) [46]	37.5	33.7	41.2
Towongo (2022) [112]	37.4	33.5	41.2
Mwanamwambwa (2021) [115]	37.0	33.3	40.7
Sserwanja et al. (2022) [99]	37.0	33.4	40.7
Housni et al. (2017) [89]	36.8	33.1	40.5
Tessema et al. (2023) [72]	37.3	33.6	41.1
Dibabu et al. (2021) [73]	37.2	33.5	41.0
Hassen et al. (2019) [100]	37.0	33.3	40.8
Betty et al. (2018) [113]	37.6	33.9	41.4
Mbaaka et al. (2019) [116]	37.3	33.6	41.1
Musaa et al. (2022) [84]	37.1	33.4	40.8
Innocent (2021) [42]	37.2	33.5	41.0
Rahantalle et al. (2017) [102]	37.0	33.3	40.7
Tolefac et al. (2017) [47]	37.1	33.3	40.8
Abera et al. (2023) [74]	37.3	33.5	41.0
Sinyange (2016) [117]	37.5	33.7	41.3
Alemu et al. (2018)| [25]	37.2	33.4	40.9
Tungaraza et al. (2022) [107]	37.4	33.7	41.2
Anaba et al. (2022) [78]	37.5	33.7	41.2
**Combined**	**37.1**	**33.3**	**41.0**

## Contributing factors of first-trimester ANC in Africa

**Fig. 6:**
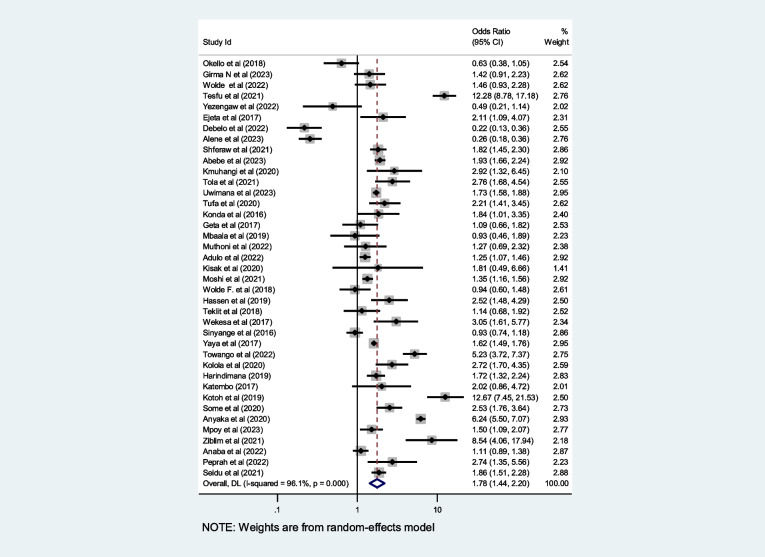
Pooled odds ratio for the association between education and first-trimester ANC contact in Africa

**Fig. 7:**
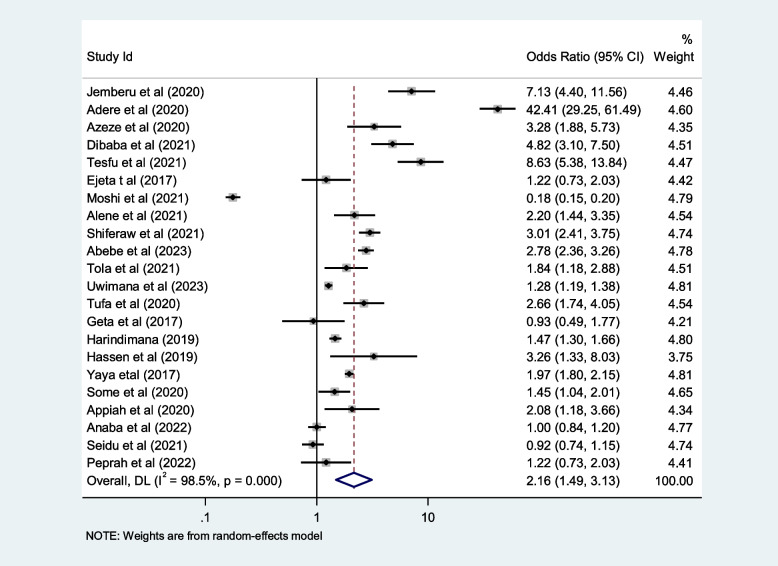
Pooled odds ratio for the association between residence and first-trimester ANC contact in Africa

**Fig. 8:**
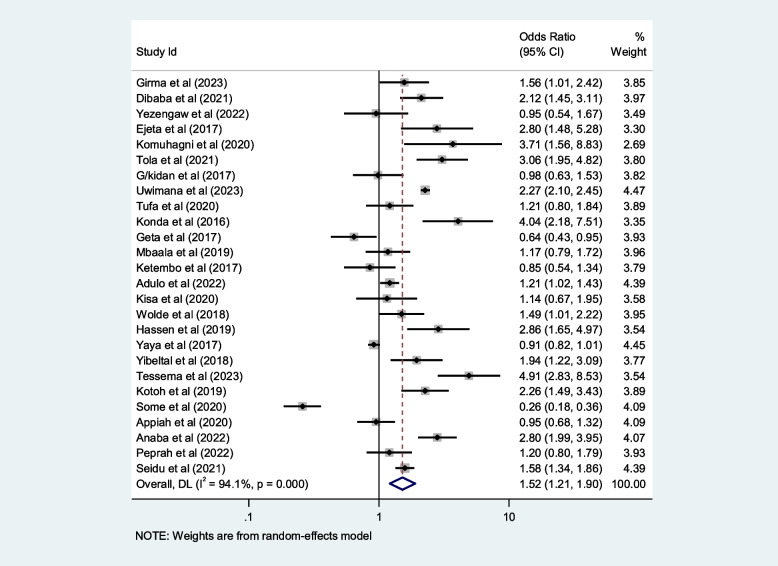
Pooled odds ratio for the association between age and first-trimester ANC contact in Africa

**Fig. 9:**
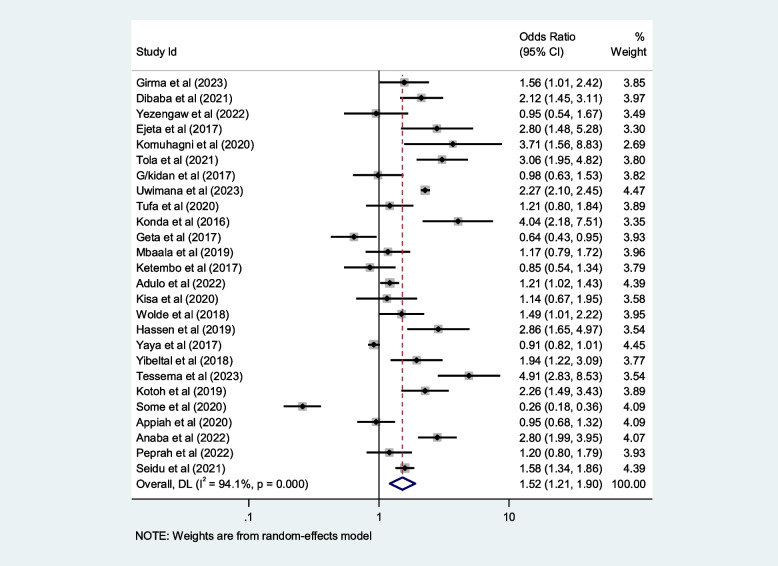
Pooled odds ratio for the association between planned pregnancy and first-trimester ANC contact

**Fig. 10:**
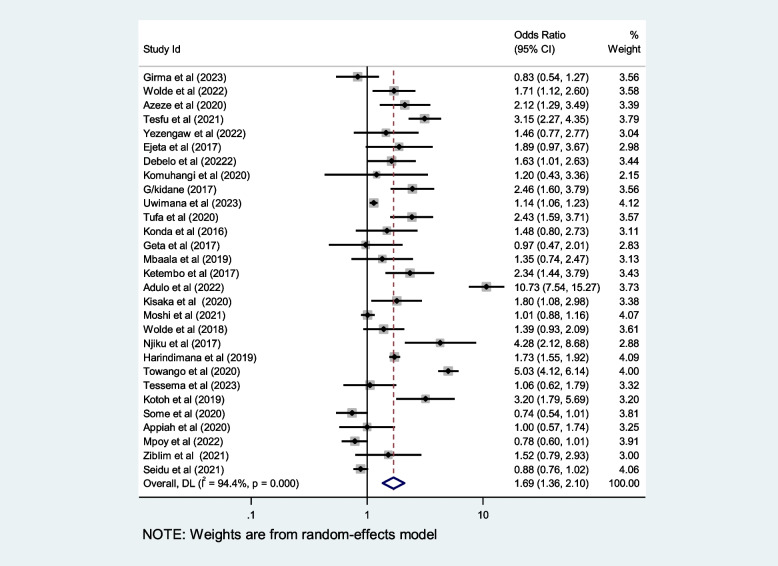
Pooled odds ratio for the association between occupation first-trimester ANC contacts in Africa

**Fig. 11:**
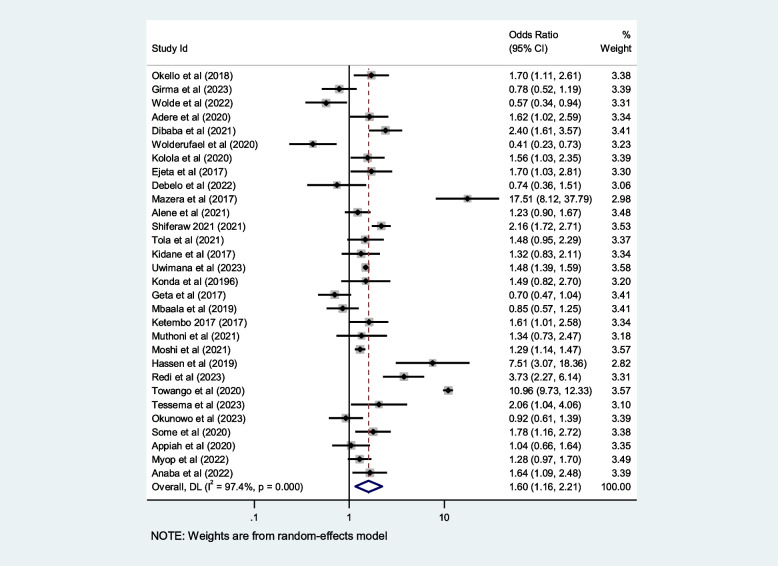
Pooled odds ratio for the association between parity and first trimester ANC contact in Africa

**Fig. 12:**
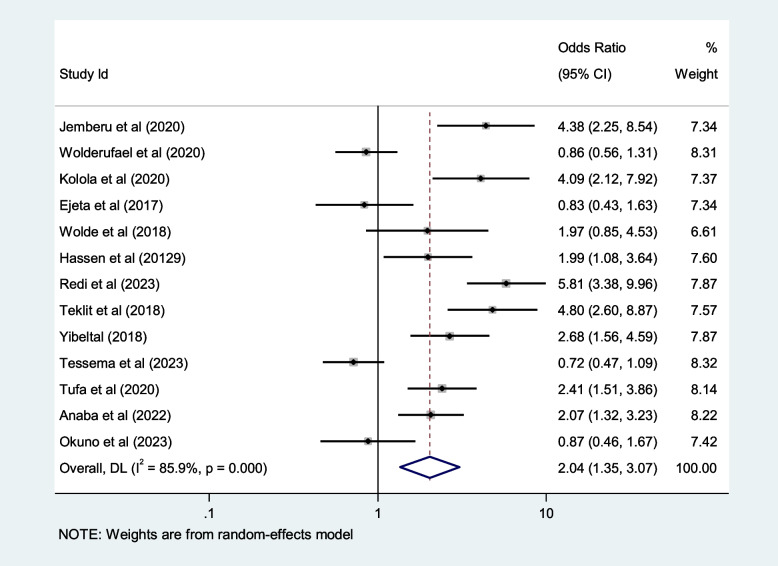
Pooled odds ratio for the association between ANC knowledge and first-trimester ANC contact in Africa

**Fig. 13:**
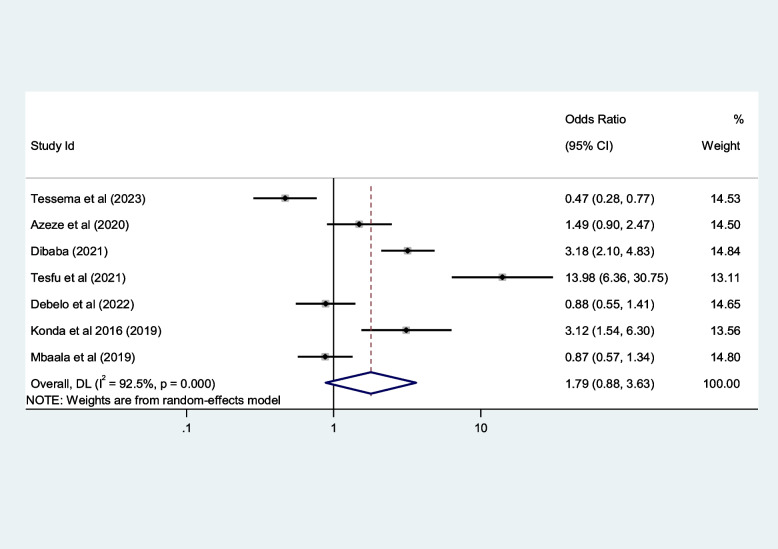
Pooled odds ratio for the association between women’s autonomy and first-trimester ANC contact in Africa

**Fig. 14:**
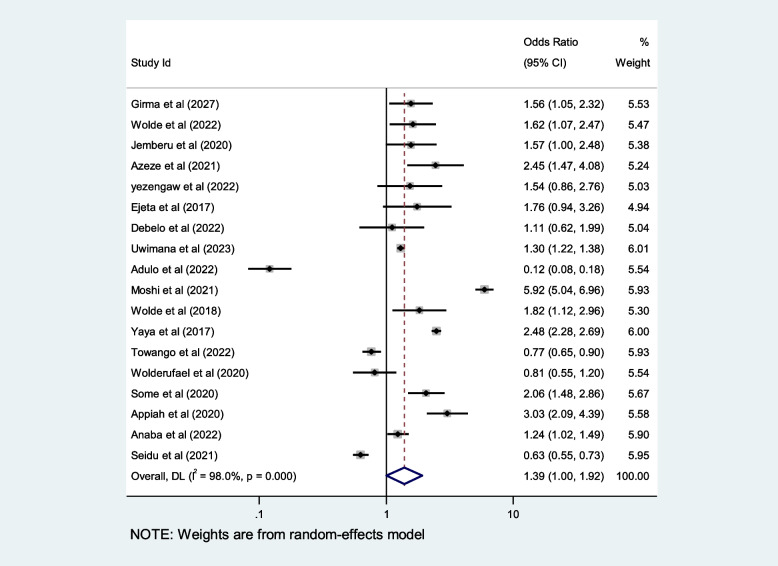
Pooled odds ratio for the association between economic status and first-trimester ANC contact in Africa

**Fig. 15:**
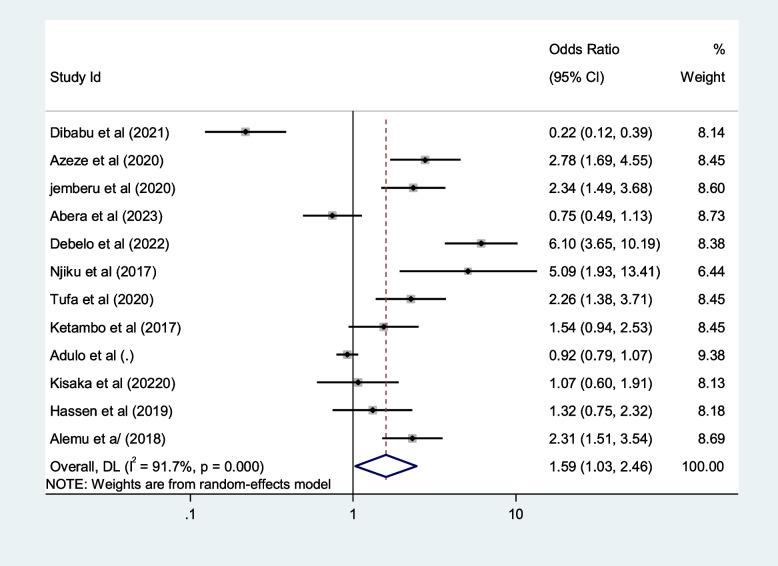
Pooled odds ratio for the association between distance from health facilities and first-trimester ANC contact in Africa

We included ten selected variables to identify relationships with first-trimester ANC contacts in Africa. Of these nine variables, namely education status, maternal age, residence, distance from health facilities, knowledge regarding timing of ANC, pregnancy status and working status (employed/working outside home) were identified as contributing factors of first-trimester ANC contact in Africa. Women aged $<$ 25 years and those who attained secondary or higher education levels were 50% and 80% more likely to initiate first-trimester ANC, respectively (OR: 1.5; 95% CI: 1.2–1.9; *P* < 0.001; *I*^*2*^: 93.9%) and (OR: 1.8; 95% CI: 1.4–2.2; *P* < 0.001**;** *I*^*2*^: 96.1%). Women who were employed and had good knowledge of the timing of ANC were 1.7 and 2.2 times more likely to initiate ANC contact in the first trimester of pregnancy, respectively (OR: 1.7; 95% CI: 1.4–2.1; *P* < 0.001; *I*^*2*^ = *94.6*%) and (OR: 2.2; 95% CI: 1.2- 2.4; *P* < 0.001;* I*^*2*^ = *86.0%*), than their counterparts. The first-trimester ANC contact rate was highest among women who had resided in urban areas compared to their counterparts (OR = 2.2; 95%CI: 1.5–3.1; *P* < 0.001; *I*^*2*^ = 98.5%). The probability of initiating ANC contact in the first trimester was 110% higher among women who had planned pregnancy (OR = 2.1; 95%CI: 1.5–2.7; *P* < 0.001; I^2^ = 95.5%). Women who were living near health facilities were 60% more likely to initiate ANC in the first trimester of pregnancy than women who were living far from health facilities (OR: 1.6; 95% CI: 1.1- 2.4; *P* < 0.001; I^2^ = 91.7%*).* The odds of first-trimester ANC contact were 60% higher for women who were primiparous than for with women who had given birth two more times (OR = 1.6; 95% CI: 1.2–2.4; *P* < 0.001; I^2^ = 97.4%). Moreover, this study also showed that women who had the highest family income were approximately 40% more likely to initiate first-trimester ANC contact, than their counterparts (OR: 1.4; 95% CI: 1.1–1.9; *P* < 0.001; I^2^ = 98.0%). Details of the contributing factors for ANC contact in the first trimester of pregnancy are described in Table 5 and Figs. 6, 7, 8, 9, 10, 11, 12, 13, 14 and 15.

**Table 5:** Contributing factors of first-trimester ANC contact in Africa, 2023

**Factors**	**Comparison**	NS*	**SS**	OR(95%CI)	**I**^**2**^**%**	**Egger test**	
1. Education	Illiterate vs Literate	39	77,614	1.8[1.4,2.2]	96.1	0.4	Figure 6
2. Residence	Urban vs rural	22	59,313	2.2[1.5,3.1]	98.5	0.8	Figure 7
3. Age	$<$ 25 vs $>$ 25 years	26	16,015	1.5[1.2, 1.9]	94.1	0.7	Figure 8
4. Pregnancy intention	Planned vs Not	21	42,365	2.1[1.5,2.7]	95.5	0.1	Figure 9
5. Occupation	Employed vs not-employed	29	60,933	1.7[1.4,2.1]	94.4	0.1	Figure 10
6. Parity	Primi vs other	30	46,619	1.6[1.2, 2.4]	97.4	0.2	Figure 11
7. Knowledge of the timing of ANC	Good vs poor	13	5263	2.2[1.4,3.5]	85.9	0.2	Figure 12
8. Autonomy	Women vs other	7	6911	1.8[0.9,3.6]	92.5	0.1	Figure 13
9. Income	Highest vs others	18	54,813	1.4[1.1,1.9]	98.0	0.2	Figure 14
10. Distance from health facilities	$\le$ 1 h vs $>$ 1 h	13	7332	1.6[1.1,2.4]	91.7	0.9	Figure 15

## Discussion

In this study, only 37.15% of women received first-trimester ANC contact in Africa andthis varies, although not significantly, across nations and regions. The percentage of countries with first-trimester ANC care coverage ranged from 13.7% in South Africa to 69.1% in Liberia. Even though some African countries performed better than others in the first three months of ANC engagement, the issue needs to be addressed urgently addressed if the rate is to increase significantly. This variation could be attributed to a variety of factors, including the volume of research conducted, cultural and socioeconomic differences between the nations, and inequalities in access to maternal healthcare facilities.

The statistic presented above for this study related to first-trimester ANC contact was similar to that reported in studies conducted in Afghanistan (33.1%) [122] and SSA (38.0%) [22]. However, it was lower than that of a study conducted in 54 low- and middle-income countries, with 69.1% in Central and Southern Asia, 63.5% in Eastern and Southeast Asia, 68.1% in Latin America, and the Caribbean and 54.6% in Northern America and Western Europe [123]. It was also lower than figures from other studies conducted in South Asia (59.5%) [124], India (69.3%) [125], Bangladesh (43.0%) [126], the United Kingdom (79.2%) [127], Saudi Arabia (75%) [126], Vietnam (75.2%) [128] and Malaysia (71.8%) [129]. This lower percentage may be attributed to sociodemographic variables or ineffective or inadequate strategies carried out in Africa by the relevant authorities. The whole community is impacted by the need to improve maternal healthcare services. However, there is evidence that antenatal contact can be started earlier with community involvement [130].

First-trimester ANC contact was substantially associated with younger women ($<$ 25 years) in this study, which is in line with what was found in the United Kingdom [131] and Vietnam [128]. In addition, primiparous women were more likely to start ANC contact in the first trimester. This conclusion is consistent with research conducted in Pakistan [132] and Myanmar [133]. The likelihood of beginning ANC in the first trimester rises because young women and primiparous lack adequate experience with pregnancy; they are either extremely eager to learn about the status of their pregnancy or very fearful of the situation and believe that early initiation of ANC is essential to mitigate their fear. However, research conducted in low-income areas of southern Asia [124] and sub-Saharan Africa(SSA) [22] found that first-trimester ANC contact was linked with a maternal age of 25 years and above. Higher parity was also associated with first-trimester ANC contact, according to research from southern Asia [124] and the UK [127]. This disparity might be the result of sociocultural variations. In addition, it might have been caused by the grouping of the categories. In this analysis, we categorized age and parity into two groups, which is contrary to the methodology in the aforementioned studies. They classified into more than two age and parity groups and compared one group with more than two groups.

Living in an urban area was significantly associated with the initiation of first-trimester ANC contact in this study, a finding that is supported by studies conducted in Bangladesh [126], low-resource settings [123], Myanmar [133] and SSA [22]. One explanation could be that urban women are more likely to obtain information about maternal healthcare services, which leads to an increase in ANC contact during the first trimester. Compared to less educated women, educated women are more likely to start antenatal treatment during the first trimester of pregnancy. Research from Bangladesh [126], Myanmar [133], Nepal [134], the United Arab Emirates [135], Pakistan [132] and low-resource setting countries [123] supports this finding. Women who are better educated have greater access to and better knowledge of maternity healthcare services. In addition, educated women can evaluate and comprehend the risks of pregnancy for both themselves and their unborn baby. They encounter a distinct message regarding maternal healthcare services as well, and they are able to comprehend the knowledge they have learned. Thus, educated women have quicker access to healthcare than uneducated individuals. Unfortunately, maternal education coverage is still low in low-resource settings for a variety of reasons. Improving maternal healthcare service utilization depends heavily on expanding universal education and maternal education in low-resource environments.

Consistent with studies conducted in Malaysia [129], Myanmar [133] and SSA [22], the results of this meta-analysis revealed that women who planned their pregnancies had higher rates of first-trimester ANC contact than women whose pregnancies were unplanned. This finding might be explained by the fact that women who planned their pregnancies were more likely to prioritize having a healthy pregnancy, which led to the timely adoption of first trimester ANC contact.

According to this analysis, women were also more likely to start first-trimester ANC contact if they resided close to healthcare facilities. Women who lived close to these facilities may have been encouraged to go there facilities for treatment prevent travel difficulties and delays. This finding is similar to those from studies in SSA [22]. First ANC contacts were also made by pregnant women who were knowledgeable about when it the best time to engage in it, which is in line with studies from Afghanistan [122] and Malaysia [129]. Women may not be well informed about the significance of antenatal booking, which could account for a lack of understanding about this concept.

This meta-analysis found a strong correlation between ANC contact during the first-trimester of pregnancy and having a high household wealth level. Research from Nepal [134], Pakistan [132], low-resource settings [123] and SSA [22] discovered that women in the highest family wealth quintile were more likely to initiate first-trimester ANC contact, supporting this conclusion. Numerous studies [136–139] have shown that it is a virtually universal truth that more healthcare services are used by those with the highest socioeconomic standing. Women who were working were significantly more likely to have first-trimester ANC contact, a conclusion that is consistent with that of Saudi Arabian research [140]. This is likely a result of the fact that working women have greater access to maternal and other healthcare services due to their ability to afford transportation and other associated costs.

## Limitations of the study

One of the study’s limitations is that some variables were divided into two categories from a larger number of categories, which obscured the impact of each category on the outcome variable. Recall bias may not have been completely eliminated from the research, as almost all of the studies included in the review were cross-sectional in design, and it is possible that the outcome variable was under- or overestimated. Another drawback of this study is that only ten variables were examined to determine their influence on the outcome variable.

## Conclusion

This meta-analysis revealed that a low proportion of women in Africa initiate first-trimester ANC contact. To increase this proven intervention to enhance maternal, perinatal and neonatal health, each country’s government in Africa should strengthen existing strategies or design appropriate innovations to enhance first-trimester ANC. Each government and nongovernmental organization (NGO) working on maternal health improvement in Africa should focus on increasing the education level of women and disseminating appropriate information to rural women living far from health facilities, women who have low economic status, and multiparous and older women. This finding could help policymakers and medical professionals design appropriate interventions. We advise other researchers who are interested in conducting similar trials to take other factors into account.

### Supplementary Information

**Additional file 1.** Search terms summary.

**Additional file 2.** Quality appraisal of included study.

## Data Availability

All relevant data are within the paper and its Supporting Information files.

## Abbreviations

ANC: Antenatal care
CI: Confidence interval
LMICs: Low- and middle-income countries
OR: Odds ratios
SBA: Skilled birth attendance
SSA: Sub-Saharan Africa
WHO: World Health Organisation
