# Supplementary material for: First trimester antenatal care contact in Africa: a systematic review and meta-analysis of prevalence and contributing factors
Source: BMC Pregnancy Childbirth. 2023 Oct 19;23:742. doi: 10.1186/s12884-023-06034-1 (PMC10585910; doi:10.1186/s12884-023-06034-1)
Supplement: Supplementary file 1 — Additional file 1. Search terms summary. [file 12884_2023_6034_MOESM1_ESM.docx]

| **PubMed search history** | | | | |
| --- | --- | --- | --- | --- |
|  | Concept | | **Search detail** | **Results** |
| Concept 1 map | Early ANC visit | Keyword |  |  |
|  |  |  | "Early antenatal care"[tw] OR "first antenatal care visit"[ tw] OR "early antenatal care initiation"[tw] OR “Antenatal care booking”[tw] OR “first trimester antenatal care”[tw] OR “Antenatal care”[tw] | 12,790 |
|  |  | Mesh term | “prenatal care”[Mesh] | 32,177 |
| Concept 3 map | Associated factors | Keyword | "Associated factors" [tw] OR "determinants"[tw] OR "Predictors"[tw] OR "Factors contributing"[tw] OR "correlates"[tw] | 722,368 |
|  |  |  |  |  |
| Concept 3 map | Pregnant women | Keyword | "Pregnant women"[tw] “pregnant women”[tw] | 119,729 |
|  |  | Mesh term | "Pregnant Women"[MeSH Terms] | 13,946 |
| Concept 4 map | Africa | Keyword | Africa [tw] | 192,619 |
|  |  | Mesh term | "Africa"[MeSH Terms] | 321,106 |
| Concept 5 map | Prevalence | Keywords | Prevalence[tw] or Magnitude[tw] OR Proportion[tw] | 1,556,069 |
|  |  | Mesh term | "Prevalence"[Mesh] | 340,613 |
| (("Early antenatal care"[Text Word] OR "first antenatal care visit"[Text Word] OR "early antenatal care initiation"[Text Word] OR "Antenatal care booking"[Text Word] OR "first trimester antenatal care"[Text Word] OR "Antenatal care"[Text Word] OR "Prenatal Care"[MeSH Terms]) AND ("Associated factors"[Text Word] OR "determinants"[Text Word] OR "Predictors"[Text Word] OR "Factors contributing"[Text Word] OR "correlates"[Text Word]) AND ("Prevalence"[Text Word] OR "Magnitude"[Text Word] OR "Proportion"[Text Word] OR "Prevalence"[MeSH Terms]) AND ("pregnant women"[Text Word] OR "pregnant women"[MeSH Terms]) AND ("Africa"[Text Word] OR "Africa"[MeSH Terms])) AND | | | | 280 |
| **Google Scholar** | | | | |
| Timely OR late initiation of ANC in Africa | | | | 260 |
| **Through Other sources** | | | | 16 |

((ffrft[Filter]) AND (2016/1/1:2023/3/9[pdat]) AND (English[Filter]))

| **Cochrane Search results(Filter applied:1/1/2016-3/9/2023)** | | |
| --- | --- | --- |
| ID | Search Hits | **Results** |
| #1 | ("Early antenatal care"):ti,ab,kw (Word variations have been searched) | 5 |
| #2 ("Associated factors" OR "determinants" OR "Predictors"):ti,ab,kw (Word variations have been searched) 304637 ("Associated factors" OR "determinants" OR "Predictors"):ti,ab,kw (Word variations have been searched) 304637 | ("first antenatal care visit"):ti,ab,kw (Word variations have been searched) | 35 |
| #3 | (“Antenatal care booking”):ti,ab,kw (Word variations have been searched) | 2 |
| #4 ("Level" OR "coverage" OR "Magnitude"):ti,ab,kw (Word variations have been searched) 414041 | (“first trimester antenatal care”):ti,ab,kw (Word variations have been searched) | 1 |
| #5 | (“antenatal care”):ti,ab,kw (Word variations have been searched) | 1592 |
| #6 | #1 OR #2 OR #3 OR #4 OR #5 | 1592 |
| #7 | ("Associated factors"):ti,ab,kw (Word variations have been searched) | 873 |
| #8 | (determinants):ti,ab,kw (Word variations have been searched) | 288165 |
| #9 | (Predictors):ti,ab,kw (Word variations have been searched) | 36425 |
| #10 | ("Factors contributing"):ti,ab,kw (Word variations have been searched) | 1461 |
| #11 | (correlates):ti,ab,kw (Word variations have been searched) | 97294 |
| #12 | #7 OR #8 OR #9 OR 10 OR #11 | 1322751 |
| #13 | (Prevalence):ti,ab,kw (Word variations have been searched) | 54135 |
| #14 | (Magnitude):ti,ab,kw (Word variations have been searched) | 16060 |
| #15 | (Proportion):ti,ab,kw (Word variations have been searched) | 94490 |
| #16 | #13 OR #12 OR #13 | 1330987 |
| #17 | MeSH descriptor: [Prevalence] explode all trees | 8680 |
| #18 | #16 OR #17 | 1330985 |
| #19 | MeSH descriptor: [Pregnant Women] explode all trees | 725 |
| #20 | ("Pregnant women"):ti,ab,kw (Word variations have been searched) | 18064 |
| #21 | #19 OR #20 | 18064 |
| #22 | (Africa):ti,ab,kw (Word variations have been searched) | 8328 |
| #23 | MeSH descriptor: [Africa] explode all trees | 11306 |
| #24 | #22 OR #23 | 15918 |
| #25 | **#6 AND #12 AND #18 AND #21 AND #24 with Publication Year from 2016 to 2023, in Trials** | **137** |

**CINHAL**

| Search ID# | Search terms | Result |
| --- | --- | --- |
| \| S1 \| \| --- \| | "Early antenatal care" OR "First antenatal care visit" OR "timely initiation" OR "early antenatal care initiation" OR “Antenatal care booking” OR “first trimester antenatal care” OR “Antenatal care” | 14,886 |
| S2 | (MH "Prenatal Care") | 19,743 |
| S3 | "Associated factors" OR "determinants" OR "Predictors" OR "determining factors" OR "Factor determining" OR "Factor contributing" OR "correlates" | 209,453 |
| S4 | Prevalence OR Magnitude OR Proportion | 399,376 |
| S5 | (MH "Prevalence") | 108,055 |
| S6 | (MH "Africa+") | 99,060 |
| S7 | Africa | 55,027 |
| S8 | S1 OR S2 | 22,648 |
| S9 | S4 OR S5 | 399,376 |
| S10 | S6 OR S7 | 110,280 |
| S12 | "Pregnant women" | 44,116 |
| S13 | S3 AND S8 AND S9 AND S10 AND S11 | 126 |
| **Limiters - Peer Reviewed; Published Date: 20160101-20231231; English Language: English** | | |

| **EMBASE search result** | | |
| --- | --- | --- |
| **No.** | **Query** | **Results** |
| #1 | 'early antenatal care visit' | 7 |
| #2 | 'first antenatal care visit' | 194 |
| #3 | antenatal care booking' | 44 |
| #4 | 'first trimester antenatal care' | 4 |
| #5 | 'antenatal care' | 16,015 |
| #6 | #1 OR #2 OR #3 OR #4 OR #5 | 16,015 |
| #7 | 'associated factors' | 39,945 |
| #8 | determinants | 247,736 |
| #9 | predictors | 450,087 |
| #10 | 'factors contributing' | 29,691 |
| #11 | correlates | 269,080 |
| #13 | #7 OR #8 OR #9 OR #10 OR #11 | 997,867 |
| #14 | prevalence | 1,335,461 |
| #15 | magnitude | 321,391 |
| #16 | proportion | 691,222 |
| #17 | #14 OR #15 OR #16 | 2,256,395 |
| #18 | 'prevalence'/exp | 953,485 |
| #19 | #17 OR #18 | 2,270,247 |
| #20 | 'pregnant women' | 160,810 |
| #21 | 'pregnant women'/exp | 113,781 |
| #22 | #20 OR #21 | 622,595 |
| #23 | Africa | 389,886 |
| #24 | 'Africa'/exp | 422,456 |
| #25 | #23 OR #24 | 422,456 |
| #26 | #22 OR #23 | 619,809 |
| #27 | 'Antenatal care'/exp | 180,130 |
| #28 | #6 OR #25 | 183,702 |
| **#29** | **#13 AND #19 AND #22 AND #25 AND #26 AND #28 AND [article]/lim AND [english]/lim AND [2016-2023]/py** | **379** |
