# Supplementary material for: First trimester antenatal care contact in Africa: a systematic review and meta-analysis of prevalence and contributing factors
Source: BMC Pregnancy Childbirth. 2023 Oct 19;23:742. doi: 10.1186/s12884-023-06034-1 (PMC10585910; doi:10.1186/s12884-023-06034-1)
Supplement: Supplementary file 2 — Additional file 2. Quality appraisal of included study. [file 12884_2023_6034_MOESM2_ESM.pdf]

THE JOANNA BRIGGS INSTITUTE

Additional file 2. Quality appraisal of included study

JBI Critical Appraisal Checklist for Analytical Cross Sectional Studies

Included studies	Eight JBI Critical Appraisal Checklist for Cross Sectional Studies(Yes,No,Unclear) If Yes(>=50%).....low risk(two raters).....>1=Yes 0=No/Unclear																
	Q1		Q2		Q3		Q4		Q5		Q6		Q7		Q8		Over all appraisal
	R1	R2	R1	R2	R1	R2	R1	R2	R1	R2	R1	R2	R1	R2	R1	R2	
1. Abebe et al	Y	Y	Y	Y	Y	Y	Y	Y	Y	Y	Y	Y	Y	Y	Y	Y	8
2. Abera et al	Y	Y	Y	Y	Y	Y	Y	Y	Y	Y	Y	Y	Y	Y	Y	Y	8
3. Adere et al	Y	Y	Y	Y	U	U	Y	Y	U	U	Y	Y	Y	Y	Y	Y	8
4. Adulo et al	Y	Y	Y	Y	U	N	Y	Y	Y	Y	Y	Y	Y	Y	Y	Y	7
5. Alemu et al	Y	Y	Y	Y	Y	Y	U	Y	U	Y	U	Y	Y	Y	Y	Y	6.5
6. Alene et al	U	U	Y	Y	Y	Y	Y	Y	Y	Y	Y	Y	Y	Y	Y	Y	7
7. Amoako et al	Y	Y	Y	Y	N	N	Y	Y	Y	Y	Y	Y	Y	Y	N	N	6
8. Anaba et al	Y	Y	Y	Y	Y	Y	Y	Y	Y	Y	Y	Y	Y	Y	Y	Y	8
9. Appiah et al	Y	Y	Y	Y	N	N	Y	Y	U	U	Y	Y	Y	Y	Y	Y	7
10. Azeze et al	Y	Y	U	U	Y	Y	Y	Y	Y	Y	Y	Y	Y	Y	Y	Y	7
11. Blackstone et al	Y	Y	Y	Y	Y	Y	Y	Y	Y	Y	Y	Y	Y	Y	Y	Y	8
12. Debelo et al	Y	Y	Y	Y	Y	Y	Y	Y	N	N	Y	Y	Y	Y	Y	Y	7
13. Dibabu et al	Y	Y	Y	Y	Y	Y	Y	Y	Y	Y	Y	Y	Y	Y	Y	Y	8
14. Drammeh et al	N	N	Y	Y	Y	Y	Y	Y	Y	Y	Y	Y	Y	Y	Y	Y	7
15. Ejeta et al	Y	Y	Y	Y	Y	Y	Y	Y	Y	Y	Y	Y	Y	Y	Y	Y	8
16. Ekholuenetale et al,2020	Y	Y	Y	Y	Y	Y	Y	Y	Y	Y	Y	Y	Y	Y	Y	Y	8
17. Ekholuenetale et al, 2022	Y	Y	Y	Y	Y	Y	Y	Y	Y	Y	Y	Y	Y	Y	Y	Y	8
18. Geta et al	Y	Y	Y	Y	Y	Y	Y	Y	Y	Y	Y	Y	Y	Y	Y	Y	8
19. Gidey et al	Y	Y	Y	Y	Y	Y	Y	Y	U	U	Y	Y	Y	Y	Y	Y	7
20. Girma et al	Y	Y	Y	Y	Y	Y	Y	Y	U	Y	U	U	Y	Y	Y	Y	7.25
21. Grump et al	Y	Y	Y	Y	Y	Y	Y	Y	Y	Y	Y	Y	Y	Y	Y	Y	8
22. Halima et al	Y	Y	Y	Y	Y	Y	Y	Y	U	U	Y	Y	Y	Y	Y	Y	7
23. Housni et al	U	U	Y	Y	Y	Y	Y	Y	Y	Y	Y	Y	Y	Y	U	U	5
24. Jemberu et al	Y	Y	Y	Y	Y	Y	Y	Y	Y	Y	Y	Y	Y	Y	Y	Y	8
25. Kolola et al	Y	Y	Y	Y	Y	Y	Y	Y	U	U	Y	Y	Y	Y	Y	Y	7
26. Kondale et al	Y	Y	Y	Y	Y	U	U	Y	U	U	Y	Y	Y	Y	Y	U	5.5
27. Kotoh et al	Y	Y	Y	Y	Y	Y	Y	Y	Y	Y	Y	Y	Y	Y	Y	Y	8
28. Kuuire et al	Y	Y	Y	Y	Y	Y	Y	Y	Y	Y	Y	Y	Y	Y	Y	Y	8

29. Machika et al	Y	Y	Y	U	U	Y	Y	Y	Y	Y	Y	Y	Y	Y	N	N	6
30. Manyeh et al	Y	Y	Y	Y	N	N	Y	Y	Y	Y	Y	Y	Y	Y	Y	Y	7
31. Musaa et al	Y	Y	Y	Y	Y	Y	Y	N	N	Y	Y	Y	Y	Y	Y	Y	7
32. Muthoni et al	Y	Y	Y	Y	Y	Y	Y	Y	Y	Y	N	N	N	Y	Y	Y	6.5
33. Myop et al	Y	Y	Y	Y	Y	Y	Y	Y	Y	Y	Y	Y	Y	Y	Y	Y	8
34. Njim	Y	Y	Y	Y	Y	Y	Y	Y	Y	Y	Y	Y	Y	Y	Y	Y	8
35. Nkoka et al	Y	Y	Y	Y	Y	Y	Y	Y	N	U	U	U	Y	Y	Y	Y	5.5
36. Ochako et al	Y	Y	Y	Y	Y	Y	N	N	Y	Y	Y	Y	Y	Y	N	N	6
37. Peprah et al	Y	Y	Y	Y	Y	Y	Y	Y	Y	U	U	Y	Y	Y	Y	Y	7
38. Redi et al	Y	Y	Y	Y	Y	Y	Y	Y	Y	Y	Y	Y	Y	Y	Y	Y	8
39. Samadoulougou et al	Y	Y	Y	Y	Y	Y	Y	Y	Y	Y	Y	Y	Y	Y	Y	Y	8
40. Seidu et al	N	N	Y	Y	Y	Y	Y	Y	U	U	Y	Y	Y	Y	Y	Y	6
41. Shiferaw et al	Y	Y	Y	Y	Y	Y	Y	Y	U	U	Y	Y	Y	Y	Y	Y	7
42. Some et al	Y	Y	Y	Y	Y	Y	Y	Y	Y	Y	Y	Y	Y	Y	Y	Y	8
43. Tesfu et al	U	U	U	U	Y	Y	Y	Y	Y	Y	Y	Y	Y	Y	Y	Y	6
44. Teshale et al	U	U	Y	Y	Y	Y	Y	Y	Y	Y	Y	Y	Y	Y	Y	Y	7
45. Tessema et al	Y	Y	Y	Y	Y	Y	Y	Y	Y	Y	Y	Y	Y	Y	Y	Y	8
46. Tola et al	Y	Y	U	U	Y	Y	Y	U	U	Y	Y	Y	Y	Y	Y	Y	6
47. Tolefac et al	Y	Y	Y	Y	Y	Y	Y	Y	Y	Y	Y	Y	Y	Y	Y	Y	8
48. Tufa G et al	Y	Y	Y	Y	Y	Y	Y	Y	U	U	Y	Y	Y	Y	Y	Y	7
49. Wekesa et al	Y	Y	Y	Y	Y	Y	Y	Y	Y	U	U	U	U	Y	Y	Y	6
50. Wolde et al	Y	Y	Y	Y	Y	Y	Y	Y	Y	Y	Y	Y	Y	Y	Y	Y	8
51. Wolde et al	Y	Y	Y	y	Y	Y	Y	Y	N	N	Y	Y	Y	Y	Y	Y	8
52. Yaya et al	Y	Y	Y	Y	Y	Y	Y	Y	Y	Y	Y	Y	Y	Y	Y	Y	8
53. Yezengaw	Y	Y	Y	Y	Y	Y	Y	Y	U	U	Y	Y	Y	Y	Y	Y	7
54. Ziblim et al	U	U	Y	Y	Y	Y	Y	Y	Y	Y	Y	Y	Y	Y	N	N	6
55. Okunowo et al	Y	Y	Y	Y	Y	Y	Y	Y	Y	Y	Y	Y	Y	Y	Y	Y	8
56. Aliyu et al	Y	Y	Y	Y	Y	Y	Y	Y	Y	N	N	N	Y	Y	N	N	6
57. Utuk et al	Y	Y	Y	Y	N	N	U	U	Y	Y	Y	Y	Y	Y	Y	Y	6
58. Fagbamigbe et al	Y	Y	U	U	Y	Y	Y	Y	Y	Y	Y	Y	Y	Y	Y	Y	7
59. Anyaka et al	Y	Y	Y	Y	Y	Y	Y	Y	Y	Y	Y	Y	Y	Y	N	N	7
60. Harindimana et al	Y	Y	Y	Y	Y	Y	Y	Y	Y	Y	Y	Y	Y	Y	Y	Y	8
61. Uwimana et al	Y	Y	Y	Y	Y	Y	Y	Y	Y	Y	Y	Y	Y	Y	N	N	7
62. Bagambe et al	N	N	Y	Y	Y	Y	Y	Y	Y	U	U	Y	Y	Y	Y	Y	6
63. Sserwanja et al	Y	Y	Y	Y	Y	Y	Y	Y	Y	Y	Y	Y	Y	Y	Y	Y	8
64. Innocent et al	Y	Y	Y	Y	Y	Y	Y	Y	Y	Y	Y	Y	Y	Y	Y	Y	8
65. Sserwanja et al	Y	Y	Y	Y	Y	Y	Y	Y	Y	Y	Y	Y	Y	Y	Y	Y	8
66. Hassen et al	Y	Y	Y	Y	Y	Y	Y	Y	Y	Y	Y	Y	Y	Y	Y	Y	8
67. Smith	Y	Y	Y	Y	Y	Y	Y	Y	Y	Y	Y	Y	Y	Y	U	U	7
68. Haffejee et al	Y	Y	Y	Y	Y	Y	Y	N	N	Y	Y	Y	Y	Y	N	N	6
69. Rahatamalleh et al	Y	Y	Y	Y	Y	Y	Y	Y	Y	N	N	Y	Y	Y	Y	Y	6
70. Moshi et al	Y	Y	Y	Y	Y	Y	Y	Y	Y	Y	Y	Y	Y	Y	Y	Y	8

71. Njiku et al	U	U	Y	Y	Y	Y	Y	Y	Y	Y	Y	Y	Y	Y	Y	Y	7
72. Katembo I	N	N	Y	Y	Y	Y	Y	Y	Y	Y	Y	Y	Y	Y	Y	Y	7
73. Kisaka et al	U	N	Y	Y	Y	Y	Y	Y	Y	Y	Y	Y	Y	Y	Y	Y	7
74. Tungaraza et al	U	N	Y	Y	N	U	Y	Y	Y	Y	Y	Y	Y	Y	Y	Y	6
75. Okello et al	Y	Y	Y	Y	Y	Y	Y	Y	Y	Y	Y	Y	Y	Y	Y	Y	8
76. Benah et al	Y	Y	Y	Y	N	N	U	U	N	N	Y	Y	Y	Y	Y	Y	5
77. Mazera et al	Y	Y	Y	Y	Y	Y	Y	Y	Y	Y	U	U	Y	Y	Y	Y	7
78. Komuhangi et al	Y	Y	Y	Y	Y	Y	Y	Y	Y	Y	Y	Y	Y	Y	Y	Y	8
79. Towongo et al	U	U	Y	Y	Y	Y	Y	Y	Y	Y	Y	Y	Y	Y	Y	Y	7
80. Betty et al	U	U	Y	Y	U	U	Y	Y	Y	U	U	Y	Y	Y	Y	Y	6
81. Chewe et al	U	U	U	U	Y	Y	Y	Y	Y	Y	Y	Y	Y	Y	Y	Y	7
82. Mwanamwambwa et al	Y	Y	Y	Y	Y	Y	Y	Y	Y	Y	Y	Y	Y	Y	Y	Y	6
83. Mbaala et al	Y	Y	Y	Y	U	U	Y	Y	Y	Y	Y	Y	Y	Y	Y	Y	7
84. Sinyange et al	Y	Y	Y	Y	U	U	U	U	Y	Y	Y	Y	Y	Y	N	N	5

JBI Critical Appraisal Checklist for Case-control Studies

Included studies	**Eight JBI Critical Appraisal Checklist for case-control Studies(Yes,No,Unclear)<br>If Yes(>=50%).....low risk(two raters).....>1=Yes 0=No/Unclear**																				
	Q1		Q2		Q3		Q4		Q5		Q6		Q7		Q8		Q9		Q10		Over all appraisal
	R1	R2	R1	R2	R1	R2	R1	R2	R1	R2	R1	R2	R1	R2	R1	R2	R1	R2	R1	R2	
Wolderufael et al	Y	Y	N	N	Y	Y	Y	Y	Y	Y	Y	Y	Y	Y	Y	Y	Y	Y	Y	Y	9.5
Gebrekidan et al	Y	Y	N	N	Y	Y	Y	Y	Y	Y	Y	Y	Y	Y	Y	Y	Y	Y	Y	Y	9.5
